# Role-play for medical students learning about communication: Guidelines for maximising benefits

**DOI:** 10.1186/1472-6920-7-3

**Published:** 2007-03-02

**Authors:** Debra Nestel, Tanya Tierney

**Affiliations:** 1 Department of Biosurgery & Surgical Technology, 10^th ^floor, St Mary's Hospital, Queen Elizabeth Queen Mother Building, Imperial College London, South Wharf Road Street, London W2 1NY, UK

## Abstract

**Background:**

Role-play is widely used as an educational method for learning about communication in medical education. Although educational theory provides a sound rationale for using this form of simulation, there is little published evidence for its effectiveness. Students' prior experiences of role-play may influence the way in which they engage in this method. This paper explores students' experiences with the aim of producing guidelines for maximising the benefits of role-play within this learning context.

**Methods:**

First-year undergraduate medical students participated in a role-play session as part of their communication programme. Before and after the session, students completed questionnaires. In the pre-session questionnaire, students were asked about their experiences of role-play and asked to identify helpful and unhelpful elements. Immediately after the session, students answered similar questions in relation to the role-play activity they had just completed. Descriptive statistics were used to analyse quantitative data and qualitative data was thematically analysed.

**Results:**

284 students completed evaluation forms. Although 63 (22.2%) had prior unhelpful experiences, most students (n = 274; 96.5%) found this experience helpful. Summary findings were that students reported the key aspects of helpful role-play were opportunities for observation, rehearsal and discussion, realistic roles and alignment of roles with other aspects of the curriculum. Unhelpful aspects were those that evoked strong negative emotional responses and factors that contributed to a lack of realism.

**Conclusion:**

Role-play was valued by students in the acquisition of communication skills even though some had prior unhelpful experiences. Guidelines for effective role-play include adequate preparation, alignment of roles and tasks with level of practice, structured feedback guidelines and acknowledgment of the importance of social interactions for learning.

## Background

Although role-play is regularly used to develop communication skills in medical students [1-12], there are few published papers that evaluate role-play as an educational method. Our experience of using role-play to teach students about communicating has met with mixed success. Introducing role-play to a group almost always meets with resistance and/or anxiety from some students. This is manifested in student apprehension, reporting of prior unhelpful experiences and not taking role-play seriously. Other researchers have also reported similar preconceptions to role-play. Stevenson and Sander (2002) reported that "role-play and student presentations" are the least preferred teaching method by 32% of new medical students [13]. Of these students, 75% believed it to be ineffective while 25% reported personal reasons (e.g. embarrassment) for their response.

### Role-play as a simulation methodology

Role-play is used as a training method to acquire knowledge, attitudes and skills in a range of disciplines and with learners of different ages (e.g. acquisition of language skills [14], cross-cultural training [15,16], business and human resources [17,18]. Despite its widespread use, role-play is fairly consistently defined in the education and training literature. Van Ments (1989) defines role-play as:

"... one particular type of simulation that focuses attention on the interaction of people with one another. It emphasises the functions performed by different people under various circumstances. The idea of role-play, in its simplest form, is that of asking someone to imagine that they are either themselves or another person in a particular situation. They are then asked to behave exactly as they feel that person would. As a result of doing this they, or the rest of the class, or both, will learn something about the person and/or situation. In essence, each player acts as part of the social environment of the others and provides a framework in which they can test out their repertoire of behaviours or study the interacting behaviour of the group." [19]

This definition clearly demonstrates that role-play is a form of simulation and acknowledges the importance of the social context of learning. Jones (1982) highlights this notion in role-play for learning second languages:

"In order for a simulation to occur the participants must accept the duties and responsibilities of their roles and functions, and do the best they can in the situation in which they find themselves" [14].

This interdependence in learning may prove to be a barrier for some students who for various reasons may be unfamiliar or unwilling to give of themselves in role-play [20]. Burns and Gentry (1998) describe the importance of gradual introduction of role-play as students may be unaccustomed to working experientially and that cultural barriers may impede student engagement [21].

Role-play activities can be performed in different ways. For the acquisition of patient-centred interviewing skills we tend to use the approach in which students play their role as a medical student so they are expected to perform as they would in real clinical encounters. However, there are many variations on this theme. Role-play can be fully scripted (all players act from verbatim scripts) or partially scripted (players have certain prompts – often an opening line). Alternatively, one player (e.g. patient) is given a description of their role while the other (e.g. student) is provided with their task. Players can rotate through roles within a single role-play (switching) with the intention of gaining insight into other roles or perspectives or players can be substituted at various points in the role-play by observers. Some role-play activities use role cards as a way of inserting new information into a role-play.

Maier (2002) suggests that role-play method be selected according to whether the educational goal addresses knowledge, attitudes or skills. In the acquisition of knowledge, role-plays can be valuable to observe and then discuss – the experience of the role-players themselves is less important than the opportunity to observe, understand and assimilate information. For attitude development especially that which focuses on change of affect, then role-plays should be loosely structured so that players experience emotions spontaneously. While for skills acquisition, the opportunity for repeated opportunities with feedback is critical [22].

### Educational theory

Role-play as an educational method draws on a range of theories. Experiential learning is especially important in the acquisition of skills. Kolb & Fry (1975) describe four "learning environments" in their theory of experiential learning [23] – Affectively-oriented (feeling), symbolically-oriented (thinking), perceptually-oriented (watching), behaviourally-oriented (doing). Within each environment there are two tasks. First, "grasping" which consists of concrete experiences and abstract conceptualisation. Second, "transforming" which consists of reflection and action. Learning is enhanced when learners are encouraged to use all four "environments." Structured role-play with feedback enables learners to complete both tasks in each of the four "environments."

Knowles (2005) has set out principles associated with adult learning. These have evolved since his first publications in the 1970's. They include the learner having a need to know, is self-directed, has and draws on diverse experiences, has a readiness to learn, that the learning should be problem-centred and that there is an internal motivation to learn [24]. Role-plays should be created and implemented with these principles in mind.

Schon's (1983) work on reflective practice is also relevant in role-play [25]. Based on his observations of different professional groups, Schon describes how when faced with unexpected events or problems, professionals respond by reflecting-in-action or later by reflecting-on-action. Reflection-in-action or "thinking on your feet" is a process by which a practitioner draws on a repertoire of experiences – images, ideas, actions, patterns in order to make sense of the problem confronting them. The practitioner aims to place the problem within their own frame of reference in order to seek out the best solution and anticipate its' consequences. Reflecting-on-action or "retrospective thinking" occurs after the unexpected event or problem and has particular relevance for feedback since discussion of what has taken place can help the practitioner broaden their base of experience and so extend their repertoire. Again, structured role-play enables learners to reflect both "in" and "on" action.

Kneebone (2005) describes a theory-based approach to learning clinical skills in simulation drawing on literature on expertise, supportive tailored tutoring, learning within a professional context and affective elements of learning. Using this theory he identifies criteria for evaluating simulations:

1. Simulations should allow for sustained and deliberate practice in a safe environment and that simulations ensure skills are consolidated and aligned with other curricula activity

2. Simulations should provide access to expert tutors who are available only when needed

3. Simulations should map on to real life clinical experience

4. Simulation-based learning should provide a supportive, motivational and learner-centred environment [26]

Although Kneebone describes these criteria in relation to learning clinical procedures, we argue that they are relevant to medical interviewing since communication is the core clinical skill and has many technical elements. All clinician-patient encounters have structural components – a beginning, middle and end. Although often described as "process" elements within medical interviewing, communication skills can be articulated in the same way as technical aspects of clinical procedures. Therefore, the criteria developed by Kneebone should be considered in constructing and implementing role-plays aimed at developing clinical communication.

### Role-play activities in our communication programme

First-year medical students at Imperial College participate in role-play activities. Table 1 outlines the broad content and educational methods in our first-year communication programme. The goal of the first session in which students use role-play includes developing competence in specific skills associated with medical interviewing. In tutor-led groups of approximately 30, students break into trios to perform role-play. We use a structured approach to role-play in which students rotate through the roles of interviewer, patient and observer. Potentially each role may benefit students as they are encouraged to adopt different perspectives. Although the "patient" roles vary within these activities, the "student" and "observer" tasks remain consistent. For example, the interviewer's task is to practice medical interviewing with a specific focus (Table 2). The observer's role is to identify skills demonstrated and areas of content explored as well as facilitate feedback to the interviewer using structured guidelines (Table 3). Patient roles provide an overview of key aspects of the presenting complaint and are sufficiently flexible to be played by students in our diverse cohorts (Table 4). Each role is allocated 5 minutes preparation, 5 minutes in role-play and 10 minutes in feedback. Part of the feedback process involves a brief period in which reflection on performance is encouraged and after the session, students are encouraged to write reflections. Between each role-play, the large group convenes and discusses issues that emerged in the role-plays as well as checks on the "role-play" process.

**Table 1:** Communication programme in year 1

Topics in sessions:
• Evidence for patient-centred interviewing
• Skills for communicating with patients
○ Non-verbal
○ Verbal
• Giving and receiving feedback
• Making presentations

Educational methods:
• Lectures
• Readings
• Small group discussions
• Role-play – observing, interviewing, facilitating feedback
• Interviews with simulated patients – actors and volunteers
• Videotape review
• Written reflections

**Table 2:** Task for interviewer

• You are a medical student attached to a general practice.
• The GP is running a little late and has asked you to go and talk to the next patient.
• As well as finding out why the patient has come to the clinic today (and what the patient expects from the consultation), ask some questions about background information such as the patient's family (and personal) relationships and his/her occupation.
• It can also be helpful to identify the patient's worries or concerns about the visit.
• Take care to explore all the patient's difficulties early in the interview.

**Table 3:** Task for observer

• Use the checklist to identify which skills the interviewer uses in the consultation.
• Facilitate the feedback process. The following points provide a structure for feedback.
• The following questions may be helpful in staying focused on your task and ensuring a balance.
○ **Can you briefly state how you felt during the interview**?
○ **Can you describe two aspects of the interview that worked well?**
• Observer asks the role-play patient:
○ **Can you identify two communication skills that the interviewer used that were effective?**
• Observer provides specific feedback on two skills that s/he observed worked well
• Observer asks the interviewer:
○ **Can you identify two aspects of the interview that you would do differently if you could repeat the interview?**
• Observer asks the role-play patient:
○ **Can you identify two communication skills that the student could have used to improve the interview?**
• Observer provides feedback on two skills that could have improved the interview
• Observer summarises the feedback on things that worked well and things to improve
• Interviewer receives written feedback from observer and role-play patient

**Table 4:** Patient role

• You are a 21-year-old student starting the third year of your engineering degree.
• You have just moved into a new flat with some friends.
• You had an appointment to register at a new clinic scheduled for next week. However, last night while you were in the garden of your new flat (trying to tidy up the mess) you scratched your hand on a dirty and rusty nail. The wound bled quite a bit but it stopped after you put pressure on it.
• You have arrived at the clinic and have been told that you can be seen in about 15 minutes by the practice nurse and if necessary a GP afterwards.
• The receptionist has asked if you would mind if a first year medical student interviewed you before the practice nurse sees you.
• You are worried about the wound. Think about how you would demonstrate your concerns to a "moderate" level. Remember to use non-verbal and verbal behaviours. If the interviewer acknowledges your worry – "I can see you are worried. I think that coming to the clinic and having the wound checked out is important."
• You think you might need a tetanus injection. You are terrified of injections. Do not mention this unless the medical student interviewer asks you about your worries or anxieties.
• You can't remember when you had your last tetanus injection.
• Either make up the rest of the story or be yourself.

Because the students are in their third week of medical school, tutors are unlikely to have had an opportunity to develop a relationship with them. Tutors are encouraged to use the following questions to promote discussion and reflection in the large group:

1. How many of you asked about the patient's ideas, concerns and expectations?

2. Did your group encounter difficulties with the introduction? The information gathering? The closure?

3. Did you follow guidelines for feedback? If so, were they helpful?

4. Did you experience any difficulties in role-play?

## Methods

First-year undergraduate medical students participated in the role-play session described above. Before and after the session, students completed questionnaires with closed and open-ended questions. In the pre-session questionnaire, students were asked about their prior experiences of role-play, if they were helpful (yes or no response) and then asked to identify helpful and unhelpful aspects (free text). Immediately after the session, students were asked to complete the same evaluation form that asked whether the role-play was helpful and again, helpful and unhelpful aspects but this time related to the role-play experience in this session. Qualitative data was using SPSS 14.0 for descriptive statistics. Qualitative data was thematically analysed by both authors and then key concepts identified and negotiated for agreement. The study took place in the academic year 2002/2003. Students completed the forms as part of their usual session evaluation that is not compulsory but is built into the session schedule. The study was approved within our College requirements for course evaluations and dissemination of results. Students were assured that all responses were anonymous, would be used to improve future sessions and findings would be disseminated in our professional community.

This paper reports on a questionnaire based study exploring first year medical students' experiences of role-play with a particular focus on supporting the acquisition of patient-centred interviewing skills. The study aims to identify key elements for establishing effective role-play addressing the following questions:

• To what extent do students find role-play valuable?

• What are helpful and unhelpful aspects of role-play?

## Results

### Students

284 students completed written forms before and after the session representing 88.8% of the cohort. Demographic data was not collected from students.

#### Pre-session questionnaire

One hundred and ninety-nine students (70.1%) reported experience with role-play and in a range of contexts. For example, learning about negotiation, presenting, public speaking, interviewing aspects, acting out scenarios for group discussion of ethical or other controversial issues and developing dramatic skills. Two hundred and twenty-one students (77.8%) stated that role-play was valuable for learning while the remainder (n = 63; 22.2%) reported it was not valuable.

In free text comments students reported role-play was valuable for gaining insight into their own and others' behaviours, increasing understanding of certain issues and rehearsing skills. Free text comments include:

"It (role-play) is a pleasant and practical way of learning and evaluating your capabilities."

"Can be amusing and interesting because it brings to life situations which may be encountered..."

Unhelpful experiences identified by students focused on emotional responses that impede learning (e.g. embarrassment, intimidation, anxiety).

"I found my nervousness interfered with my ability to address the issue."

Lack of realism of roles, setting and task were also cited as unhelpful.

"I find it very false and difficult particularly with people I don't know"

"You behave differently when you are being observed."

Unsuitable environments, not taking role-play seriously and unstructured, vague non-specific feedback were also considered as unhelpful.

#### Post-session questionnaire

After the session, 96.5% of students (n = 274) reported that role-play had been helpful for learning. Table 5 contains examples of a range of students' free text comments. Common themes include opportunity for rehearsal, importance of preparation, to receive feedback, to experience perspectives of patient and interviewer, to practice in relative safety (no consequence to patients) and to develop other professional skills such as giving and receiving feedback, facilitating and participating in discussion of peer's behaviours.

**Table 5:** Students' responses to being asked to describe ways in which role-play can be helpful

*Preparation*
*"The most important thing was the in-built preparation"*
*Rehearsal*
*"It enabled me to use my skills directly and assess their effects on other people..."*
*"First hand experience – to understand the difficulties in communication which are hard to get without actually doing it"*
*"It helped understand, what sort of questions provided the best responses"*
*"Enabled me to use my communication skills in a way much more relevant to the way in which I will need them in real consultations. Also, the role plays each included a psychosocial aspect which was useful to practice eliciting."*
*"It highlighted the importance of 'probing' in order to get information out of patients"*
*"It also made me realise how difficult it can be to keep an interview flowing – I'll definitely need more experience!"*
*"Good chance to try out something that looks so easy but makes you realise that it is difficult and does require practice."*
*Feedback*
*"It's good to receive constructive criticism and be made aware of your behaviour"*
*"Group discussion afterwards (as well as individual feedback) allowing us to learn from other people's experiences too."*
*Different perspectives*
*"Positive to see things from a patient's perspective"*
*"Got to experience how the patient feels as well as observing the interview, to get a more well rounded picture of a consultation."*
*"In playing the role of the patient, as well as the medical student, it enabled us to understand the patient's point of view."*
*Safety using role-play*
*"It puts you in a real-life situation where you can practice what you know but still it is a role-play so if you make mistakes it is okay"*
*Other professional skills*
*"Good method of learning and helping people feel less nervous talking in front of others."*
*"Highlighted aspects of non-verbal communication"*

Table 6 contains comments on unhelpful aspects as experienced in the session. The comments fall into similar categories as those identified in the pre-session questionnaire. That is, role-play was unhelpful when students experienced emotions of embarrassment, vulnerability and anxiety, when roles lacked realism, when students did not behave as they might in a real situation, if students had poor "acting" skills, when there was inadequate preparation, when there was uncertainty about the quality of peer feedback, lack of clarity in instructions, lack of opportunity to immediately transfer the experience to real clinical practice and a noisy environment.

**Table 6:** Students' responses to being asked to describe ways in which role-play can be unhelpful

Role-play – "acting"
*"Was not real hence some emotions were over acted, would not have been the same had it been done for real"*
*"Have to concentrate on acting – this can detract from thinking about what you are doing"*
*"Hard to get into role as patient and interviewer because they were unreal situations and I know the people in my group"*
*Lack of clarity in instructions/task*
*"Sometimes structure is poor (haven't been told enough about what to do)"*
*No opportunity for transfer of skills*
*"Can't relate to experiences learnt during role-play when communicating in real situations"*
*Unsuitable environment*
*"Too many students in one place so it was noisy and hard to concentrate"*
*Lack of realism*
*"I find it difficult to show empathy in these situations as the complaints are not genuine."*
*"You can never take it seriously as you know the people you're interviewing and so the way you act is not representative of how you would with a real patient."*
*"When you are being observed, you behave differently..."*
*"It is unrealistic as the person you're talking to doesn't have a real illness, so they will react differently to real patients"*
*Uncertainty of the quality of feedback*
*"I was unsure if the advice given by my peers was the same advice a lecturer or doctor would give, so I was unsure if their advice was reliable."*

Students' suggestions to improve role-play are reported in Table 7 and focus on personal, educational and organisational aspects of sessions although these categories overlap. Most comments address issues raised in the previous question on unhelpful aspects of role-play. More time, more opportunities for role-play (interview more than once so you can try to improve within the same session), more realistic roles, increased tutor enthusiasm, more feedback from tutors and where possible to work with students who are less well known to you.

**Table 7:** Students' responses to being asked for ways in which role-play can be made more effective

Educational
*"Should try and be as realistic as possible in order to be effective"*
*"Videotaping is very useful as can see how your own performance is seen by others"*
*"Have tutors who at least seem interested in it and take it seriously"*
*"Needs to be structured with clear aims and instructions"*
*"Personal feedback from tutor on performance during role-plays would have been useful"*
*"Time before the interview to prepare and think about the structure, questions etc, and time after to reflect, analyse and modify structure, questions of the interview to make it more effective."*
*"It is a change from usual way of learning that some may find refreshing"*
Personal
*"Much better doing it with people you don't know"*
*"Role-play changes your perspective on subjects and can open up new avenues of thinking"*
Organisational
*"Increase the number of role-plays to prepare us for real thing"*
*"Coordinate role plays with placements"*

## Discussion

Role-play was reported to be an effective means of learning communication skills. After participating in the session in this study, almost all students reported role-play as valuable compared with 70.1% at the pre-session questionnaire. Students identified helpful and unhelpful aspects of prior and current experiences with those in this session reported as overwhelmingly positively. Table 8 summarises guidelines for effective role-play based on the students' responses to pre- and post- session questionnaires. The guidelines reflect principles of adult learning as identified by Knowles et al (2005) [24]. In particular, the "need to know," "readiness to learn" and "orientation to learning (problem-centred)." These role-play guidelines also complement the criteria that Kneebone (2005) sets out to evaluate simulations [26]. To more completely meet the criteria in relation to the session described here, we would need to provide additional role-play opportunities, ensure access to expert tutors is available and make further attempts to increase realism. The guidelines also support those outlined in a recent paper by Joyner and Young (2006) that appear to be based on their extensive experience although no empirical data is presented [9].

**Table 8:** Guidelines for effective use of role-play to develop patient-centred interviewing skills

• State clear aims and objectives about task and roles
• Create roles that reflect real experiences and appropriate levels of challenges
• Relate the role-play to the broader contexts in which students are learning
• Acknowledge potential difficulties in role-play
• Emphasise the importance of social interactions for learning
• Provide sufficient time for preparation for roles
• Highlight benefits from playing all roles
• Use structured feedback guidelines – explore interviewers' feelings, identify effective skills and those that require development, seek feedback from interviewer and "patient", achieve a balance in what ahs worked and what needs development
• Respond to student preferences for working with friends
• Write reflections on the experience
• Ensure tutors are enthusiastic
• Provide opportunities for debriefing
• Summarise experiences
• Use audiovisual recording devices for playback

In relation to experiential learning theory, our session guidelines in which students are encouraged to consider each orientation of the Kolb and Fry's (1975) four learning environments – thinking, feeling, watching and doing – may explain why the role-play experience in this study was valued [23]. With small group feedback and large group post-interview discussions each of these orientations is explored.

We believe that the structured feedback process together with the process of completing the questionnaire and reflecting on role-play as an educational method may benefit students. Structured feedback directed students to think about what had taken place in each role-play as well as the value of role-play before and after participating in the session. This prompted students to draw on their prior experience – an important component of adult learning as well as promoting reflection-on-action. Even though we are working with trainee rather than fully fledged professionals we aim to instill the notion of reflection-in-action by actively prompting students to analyse what they have done in their role-play and how this experience relates to their own and others' experiences.

Our results differed to Stevenson and Sander (2002) with fewer students in our cohorts thinking role-play unhelpful – even at the pre-session questionnaire [13]. More information about individual students as well as cohorts may provide clarity. However, caution should of course be exercised in extrapolating results to all students in all medical schools.

The importance of social interactions in role-play are not always emphasised in this methodology and may be a significant reason why some students fail to engage. The role-play guidelines draw attention to the need for social exchange between participants in order to have successful role-play.

Like most educational methods, role-play on its own probably contributes only a little to the development of patient-centred interviewing skills. However, as part of the broader communication programme at Imperial College that uses a wide range of methodologies addressing knowledge acquisition, attitude and skills development, role-play appears to be beneficial. For this reason it is difficult and in some ways unreasonable to try to evaluate the impact of singular educational methods. It is also important to recognise that students learn in different ways and that role-play may be a preferred method for students who learn through concrete experiences.

We explored students' prior experiences as well as those from the session in order to differentiate previous and current experience. There was little evidence that students' prior experiences influenced the way in which students responded to the current session. However, we believe this was related to the investment at the beginning of the session in completing questionnaires and acknowledgement of prior difficulties together with other features of the session (e.g. peer learning, structured feedback etc).

### Limitations

The study formed part of the communication programme and so students' responses may have been biased with students providing socially desirable responses. However, medical students at our school seem able to be honest and direct in their evaluation of educational experiences. The responses were only gathered from first year students and may not reflect those of students who are further through medical education, graduates or students in other medical schools. Data on age, gender and cultural differences of students was not collected in this study but may have provided further insight. However, College records show that this cohort closely resembles others in our medical school (over the past 5 years) – most are direct entrants from school, slightly more females than males and a very broad cultural base.

We asked students a closed-ended question about helpfulness of role-play and so forced students to make a clear directional choice. It may have been more helpful to ask students about their level of agreement with a statement about the value of role-play.

### Future research

Investigating the impact of role-play on actual learning is an obvious area to explore since we focused our study on students' perceptions of learning. However, as outlined above, this is often conceptually unsound and methodologically difficult. Future research could explicitly test these guidelines in a range of practices. It would also be valuable to focus research on why some students continue to fail to engage in role-play. Eliciting the views of teachers in facilitating role-play work would also provide an additional perspective.

## Conclusion

Although role-play is widely used as a learning activity in communication and interpersonal skills training, students do not always find it helpful. This study explored students' prior experiences in role-play in an effort to identify, acknowledge and deal with some of these difficulties in a communication programme that relies heavily on role-play as a learning activity. The results identify elements for consideration when using role-play with novice students for developing communication skills. We have provided a theoretical foundation for the use of role-play as an educational method in the broader context of simulations. Future research should investigate the relevance of these guidelines for more experienced students and practitioners and in different contexts.

## Competing interests

The authors do not have competing interests (financial or non-financial) in undertaking or publishing this paper.

## Authors' contributions

DN devised the study, analysed the data and wrote the paper. TT analysed the data and revised the paper. Both authors read and approved the final version.

## Pre-publication history

The pre-publication history for this paper can be accessed here:


